# Elevated Eosinophil Count Following Pembrolizumab Treatment for Non-Small Cell Lung Cancer

**DOI:** 10.7759/cureus.16266

**Published:** 2021-07-08

**Authors:** Angel R Baroz, Isa Mambetsariev, Jeremy Fricke, Rebecca Pharaon, TingTing Tan, Trilokesh Kidambi, Karamjeet S Sandhu, Marianna Koczywas, Ravi Salgia

**Affiliations:** 1 Medical Oncology and Therapeutics Research, City of Hope National Medical Center, Duarte, USA; 2 Medical Oncology and Therapeutics Research, City of Hope National Medical Center, Newport Beach, USA; 3 Department of Medicine, Division of Gastroenterology, City of Hope National Medical Center, Duarte, USA; 4 Department of Hematology and Hematopoietic Cell Transplantation, City of Hope National Medical Center, Duarte, USA

**Keywords:** heme-related iraes, immune-checkpoint inhibitors, (pd-l1), metastatic non-small cell lung cancer, immune markers

## Abstract

Immune-related adverse events (IRAEs) are a common yet problematic phenomenon in patients who are treated with immune checkpoint inhibitors (ICIs). Current research efforts have explored the exact pathophysiology of IRAEs in the clinical setting. However, a rare subset of IRAEs that is less highlighted and may cause detrimental effects are hematological IRAEs (heme-IRAEs). Of note, immune-induced eosinophilia itself is a heme-IRAE that is worthy of further investigation.

In this report, we present two cases of advanced staged non-small cell lung cancer (NSCLC) treated with single-agent pembrolizumab, and who subsequently sustained markedly elevated eosinophil counts (EEC) on laboratory findings. The two patients were Caucasian and both were diagnosed with NSCLC, although with differing histologies: a 76-year-old male with adenocarcinoma and a 66-year-old female with squamous cell carcinoma. Programmed death-ligand 1 (PD-L1) expression was detected via immunohistochemistry (IHC) and molecular tumor profiling did not show any actionable oncogenic mutations. Both patients were treatment-naïve and received pembrolizumab as first-line systemic therapy. The male patient, a former heavy smoker, underwent 18 months of pembrolizumab treatment before high eosinophil counts and was diagnosed with immunotherapy-related apoptotic colopathy after colonoscopy. Following pembrolizumab discontinuation, he remains under surveillance with good disease control and does not show any ongoing symptoms. The female patient, a never-smoker, underwent 15 cycles of pembrolizumab before the discontinuation of the treatment after consistently high levels of eosinophil counts. Both patients were treated with systemic corticosteroids after the discontinuation of immunotherapy, and their eosinophil levels returned to normal values. However, the female patient declined any further therapy and expired 24 months after the discontinuation of immunotherapy.

Immune-induced eosinophilia is a rare event and reported in only 2.9% of NSCLC cases. Outcomes in the two patients differed, indicating that further research related to eosinophilia and its causes in the context of varying histologies and clinical profiles of patients is warranted.

## Introduction

Advancement in the treatment for non-small cell lung cancer (NSCLC) patients has grown beyond broad-based systemic chemotherapy procedures. Oncological decision-making has made great strides towards precision medicine where therapy depends on the presence of driver mutations identified via next-generation sequencing, fluorescence in situ hybridization (FISH) assays, and immunohistochemistry (IHC) indicated for targeted therapy [1-3]. However, lung cancer is a complex and heterogeneous disease where actionable alterations such as epidermal growth factor receptor (EGFR), anaplastic lymphoma receptor tyrosine kinase (ALK), and proto-oncogene tyrosine-protein kinase ROS (ROS1) are not present in a number of patients [4]. Patients who lack driver alterations are eligible for immune checkpoint inhibitors (ICIs) that target either programmed cell death protein (PD-1) or programmed death-ligand 1 (PD-L1) to induce antitumor activity and stimulate disease control using the innate immune system [5]. Factors that affect immunotherapy treatment include the level of PD-L1 expression, the extent of disease, and histology [6,7]. More recently, ICIs have become standard therapy for first-line use in patients with advanced NSCLC, either alone [8-11] or in combination with chemotherapy [12-15].

ICIs can often result in adverse events, also called immune-related adverse events (IRAEs), that may impact the outcome of a patient’s therapy and overall disease burden. The most common systems where IRAEs occur in NSCLC are the lungs, endocrine system, skin, gastrointestinal tract, and musculoskeletal systems [16]. However, a new class of hematological IRAEs (heme-IRAEs) are a rare and potentially severe subset of IRAEs, and they have recently garnered considerable attention [17-20]. Delanoy et al. have provided a comprehensive report of heme-IRAEs in a large cohort of 948 patients treated with anti-PD-1 or anti-PD-L1 monoclonal antibodies. Among the cohort, only 35 patients (3.6%) had heme-IRAEs related to ICIs, and neutropenia, autoimmune hemolytic anemia, and immune thrombocytopenia were the most common types of heme-IRAEs (each in nine patients) [17]. However, the extent of heme-IRAEs has not been fully characterized, and while PDGFRA, PDGFRB, or FGFR1 rearrangements have been associated with myeloid neoplasm-induced eosinophilia, these alterations were not detected in our two cases. Only a handful of studies have explored the relationship between regular blood draws and hematological IRAEs such as thrombotic thrombocytopenic purpura [21], but interestingly, a marked increase in eosinophil count has been rarely explored as a correlative marker with immunotherapy [18]. In this study, we present two patients with varying onset times of eosinophilia induced by pembrolizumab (anti-PD-1 antibody) administration.

## Case presentation

Patient A

**Figure 1:**
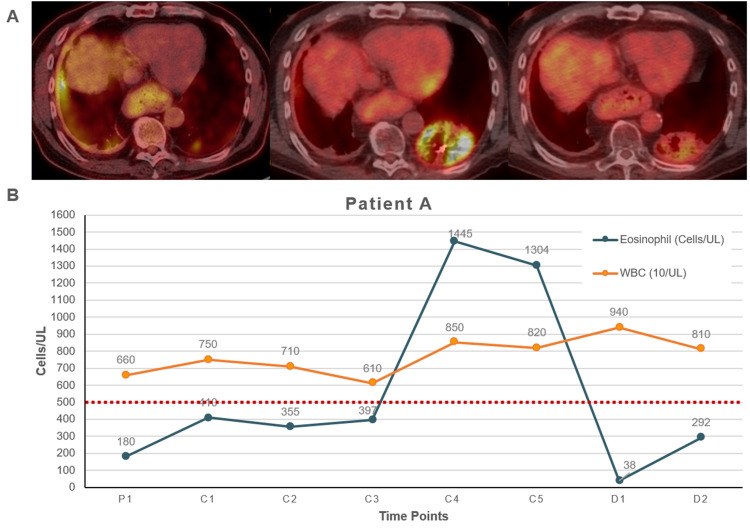
Diagnostic images and the absolute eosinophil count for patient A while on pembrolizumab A. PET demonstrating hypermetabolic activity at the pleural lining of the right lung base and a pulmonary nodule at the left lung base prior to pembrolizumab, the treatment response to pembrolizumab after discontinuing therapy, and the significant pneumonitis in the posterior left lower lobe from XRT and its partial resolution. B. Patient A absolute eosinophil and white blood cell count during pembrolizumab therapy displaying elevated counts at C4 and C5. The red line indicates the upper threshold of normal for eosinophil count PET: positron emission tomography; P: cycle at the previous institution; C: cycle; D: lab draw after discontinuation

A 76-year-old Caucasian male with multiple medical comorbidities including previously excised malignant rectal tumor (pathologic staging: T1aN0M0), hypertension, diabetes, diverticulitis, and chronic obstructive pulmonary disease (COPD) with a former 45-pack-year cigarette smoking history presented with a dry persistent cough that warranted chest imaging. A CT of the chest confirmed a hypermetabolic 1.2-cm spiculated nodule in the right upper lobe (RUL) of the lung without significant hilar or mediastinal lymphadenopathy. A robotically-assisted right upper lobectomy with hilar and mediastinal lymph node dissection’s final pathology revealed moderately differentiated adenocarcinoma of the lung with negative margins. Ten lymph nodes examined were negative for disease involvement, and it was specified as a pT1AN0 case at the time. Following the surgery, the patient was placed on surveillance and followed up with routine imaging. Approximately five years following diagnosis, he complained of right-sided posterior rib pain. MRI revealed abnormal signal intensity involving the right posterior ribs with cortical destruction of the fifth rib. A positron emission tomography and CT (PET/CT) scan showed the extent of involvement of over approximately 4.4 cm with a lytic lucency involving the right posterior fifth rib, hypermetabolic activity and pleural thickening at the right lung base, and a 0.7-cm avid pulmonary nodule at the left lung base (Figure 1A).

**Figure 2:**
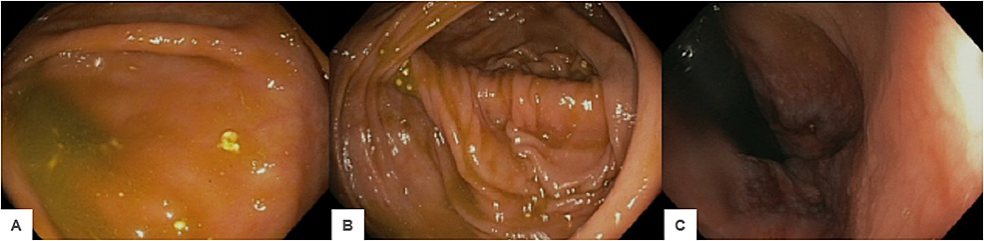
Patient A colonoscopy imaging of the appendiceal orifice (A) ileocecal valve cecum (B) and rectum (C) were normal upon visual examination. Endoscopic diagnoses confirmed no evidence of colitis, obvious large polyps, masses, or cancer

A core needle biopsy of this rib lesion showed malignant cells consistent with recurrent bronchogenic adenocarcinoma. PD-L1 IHC 22C3 pharmDx was positive with a low expression of 1%, and molecular testing revealed KRAS G12C mutation. Per the patient’s decision, he was initiated on single-agent pembrolizumab 200 mg intravenously with a good response. Approximately 18 months after the initial treatment date, laboratory results revealed high absolute eosinophils levels of 1445 cells/uL (normal range: 15-500 cells/uL) and white blood cells (WBC) count of 850 x 10/uL. The patient was administered one more dose of pembrolizumab, but absolute eosinophils levels remained consistently high at 1304 cells/uL and WBC count at 820 x 10/uL (Figure 1B). A correlative PET/CT scan confirmed post-radiation pneumonitis, associated with recent stereotactic radiosurgery (SRS) to a lesion in the left lower lung field, and pancolitis (Figure 1A). Subsequently, a colonoscopy with biopsy was performed. The appendiceal orifice, ileocecal valve cecum, and rectum appeared normal upon visual examination (Figure 2). Yet, pathology of the colon showed colonic mucosa with scattered apoptotic bodies, increased intraepithelial lymphocytosis, and eosinophils in lamina propria, consistent with immunotherapy-related apoptotic colopathy. It was decided to initiate corticosteroids and hold pembrolizumab treatment given his eosinophilia, post-radiation pneumonitis, and stable disease on scans. He is currently under surveillance with no evidence of progression of the disease since discontinuing immunotherapy six months prior.

Patient B

**Figure 3:**
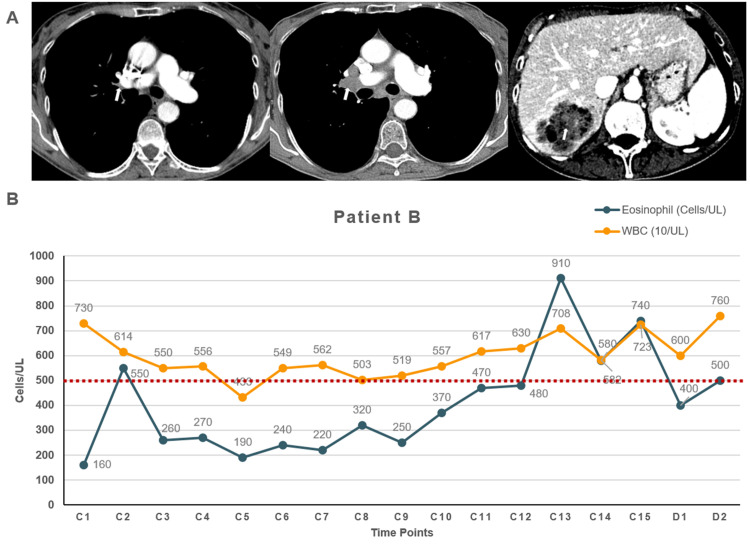
Diagnostic images and the absolute eosinophil count for patient B while on pembrolizumab A. CT demonstrating right hilar lymph node lymphadenopathy (arrow) at diagnosis and progression within the right hilar lymph node and development and progression of a segment 7 liver metastasis during pembrolizumab treatment. B. Patient B absolute eosinophil and white blood cell counts during pembrolizumab therapy displaying elevated counts at C2, C13, C14, and C15. The red line indicates the upper threshold of normal for eosinophil count CT: computed tomography; C: cycle; D: lab draw after discontinuation

A 66-year-old Caucasian female with a history of hypercalcemia, epilepsy, COPD, and a 41-pack-year smoking history presented with weight loss, worsening cough, and shortness of breath. Prompt imaging workup revealed a 3-cm speculated mass concerning for lung carcinoma. There was a contiguous soft tissue extension from the mass to the right hilum measuring 0.9 x 0.8 cm, concerning for direct tumor extension (Figure 3A). She then underwent an endobronchial biopsy and bronchoalveolar lavage of the RUL mass, and pathology confirmed moderately differentiated squamous cell carcinoma. IHC showed PD-L1 expression of 65% with 2+ intensity, and molecular testing revealed mutations in the following genes: NFE2L2 R34Q, TP53 Y220C, CCND2 amplification, CDK6 amplification, KRAS amplification, and PIK3CA amplification. A mediastinoscopy was performed, but pathology was negative for the disease. The patient declined systemic treatment at the time.

She continued to have progressively worsening cough and weight loss of more than 20 pounds. A PET/CT showed an increase in size and activity of the RUL and new possible thoracic vertebrae metastasis. The patient was started on pembrolizumab 200 mg intravenously approximately one year after initial diagnosis and therapy was well tolerated. After seven months of treatment, the patient’s absolute eosinophil count and WBC count revealed an upward trend (Figure 3B). In tandem, pembrolizumab treatment was discontinued after 10 months of therapy due to progressive disease seen on a CT of the chest, abdomen, and pelvis, which showed interval increase in the size of intrathoracic lymphadenopathy, progression of a 5.9 x 4.5-cm segment 7 liver metastasis, and new 3.6-cm simple-appearing cystic right adnexal lesion (Figure 3A). The patient underwent extensive radiation therapy and continued to take corticosteroids; however, she did not receive any further systemic treatment. She continued to decline clinically and expired 24 months after the treatment discontinuation.

## Discussion

Currently, pembrolizumab is recommended for NSCLC patients without oncogenic target mutations and with a positive PD-L1 tumor proportion score (TPS, ≥1%). As an ICI, the anti-PD1 antibody pembrolizumab turns on the immune system’s ability to recognize and target cancer cells. In the KEYNOTE-189 trial, a global placebo-controlled phase 3 study, adverse events were measured for patients with nonsquamous NSCLC with any level of PD-L1 expression in combination with pemetrexed, platinum-based drug plus either pembrolizumab or placebo [12]. IRAEs were reported in 22.7% (92/405) of patients in the pembrolizumab-combination group, and of this group, 8.9% were of grade 3 or higher. Correlative to other ICI studies, the most common IRAEs were thyroid dysfunction, pneumonitis, and colitis (10.7%, 4.4%, and 2.2%, respectively). However, heme-related IRAEs were not mentioned due to their lower frequency in occurrence. There has been an increased focus on heme-related IRAEs due to their rare yet severe implications in patient outcomes.

Both of our patients experienced delayed onset of EEC following pembrolizumab initiation (patient A: 18 months, patient B: seven months). It appeared that patient A sustained a more serious adverse effect of immunotherapy-related apoptotic colopathy. Although the tissue did not contain malignant cells, increased eosinophils in lamina propria were found in the colon. This raised concerns for eosinophils infiltrating surrounding tissues and organs. Harbaum et al. have described this phenomenon in colorectal cancers and according to them, the presence and the extent of eosinophils in surrounding tissues may be prognostic of favorable progression-free and cancer-specific survival outcomes [22]. Interestingly, patient A is still under surveillance and continues to have stable disease on radiographic scans. We cannot exclude the possibility of a primary eosinophilic gastrointestinal disorder, which could have manifested from acute colitis potentially secondary to radiation therapy [23]. The colonic biopsy revealed eosinophils within the lamina propria, which is a classic depiction of eosinophilic colitis (EC). However, pathology revealed that the eosinophils were induced by immunotherapy use. Further investigation is needed to properly diagnose primary EC vs. immune-induced eosinophilia as in the case of patient A. Patient B was not evaluated for eosinophils-infiltrating tissue, yet her disease worsened and it was deemed that she had progressive disease while on pembrolizumab. The clinical decision reached was to discontinue pembrolizumab and offer other treatments as aforementioned.

We cannot assert that the EEC in our patients was solely immune-related, and eosinophilia can be caused by a variety of sources such as asthma, allergies, tissue-invasive parasites, and various medications or drug-induced hypersensitivities. However, the two reported cases did not have any significant history of asthma, allergies, autoimmune disorders, or adrenal insufficiency. Since the patients were a male and a female each, ICI-induced eosinophilia does not appear to be gender-influenced, and both subjects were within the age range of typical lung cancer patients. Also, both patients did not have myeloid or lymphoid neoplasms with PDGFRA, PDGFRB, or FGFR1 rearrangements, which can be associated with eosinophilia, according to their molecular reports. In either patient, there was no mention of a bone marrow biopsy or any workup related to hypereosinophilic syndrome (HES) that may have explained the EEC [24]. Both cases had a relatively acute diagnosis of EEC and were carefully monitored afterward using serial blood draws. It was through the administration of corticosteroids and the discontinuation of pembrolizumab that both patients' eosinophil counts returned to normal values, which is consistent with an IRAE and which influenced their physicians’ decision to not pursue any other diagnostic intervention.

## Conclusions

This report discussed the cases of two advanced NSCLC patients treated with pembrolizumab and subsequently diagnosed with immune-related eosinophilia. Through the discontinuation of pembrolizumab and administration of corticosteroids, the patients' absolute eosinophils count returned to normal values. However, the discordant survival results of one patient achieving stable disease and the other showing progressive disease warrant further studies with an expanded cohort size, and with the characterization of clinical, molecular, and diagnostic findings in efforts to further evaluate immune-induced eosinophilia in NSCLC patients. The exact pathophysiology of immune-induced eosinophilia must also be explored to gain deeper insights into clinical approaches to the treatment of EEC.
